# Supplementary figures and images for: Foldscope: Origami-Based Paper Microscope
Source: PLoS One. 2014 Jun 18;9(6):e98781. doi: 10.1371/journal.pone.0098781 (PMC4062392; doi:10.1371/journal.pone.0098781)

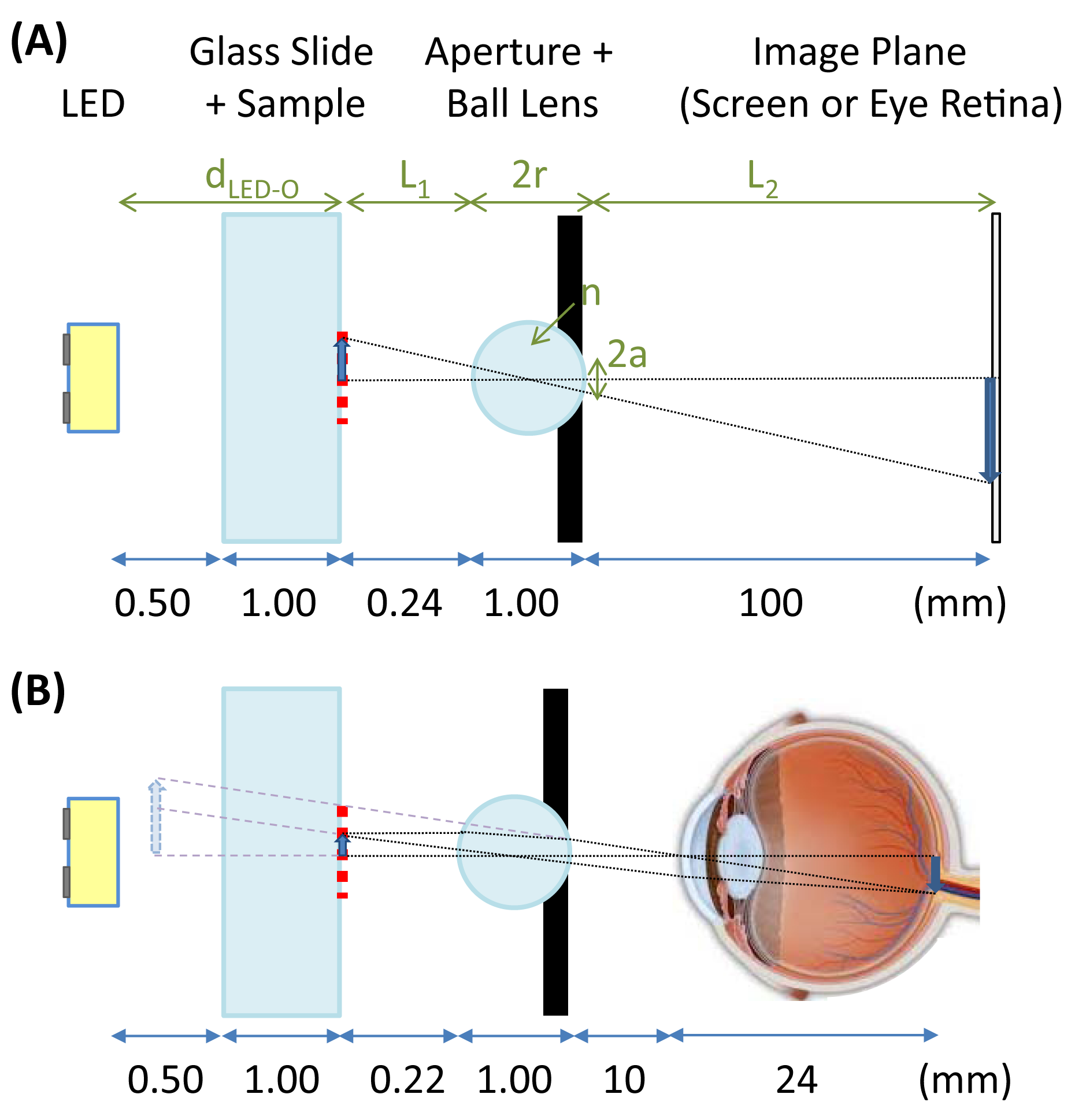

Supplement: Figure S1 — Foldscope Schematics. (A) Real image formation via projection. (B) Virtual image formation via direct observation with the eye. Note the drawings are not to scale. The indicated lengths are example values that show the versatility of this design as well as its extreme space efficiency. For example, the same system can be used for projecting or imaging simply by changing the object-lens distance by about 20 µm. Also, notice the total path length from the LED to the lens is almost an order of magnitude smaller than the size of the human eye. (TIF) [file pone.0098781.s001.tif]

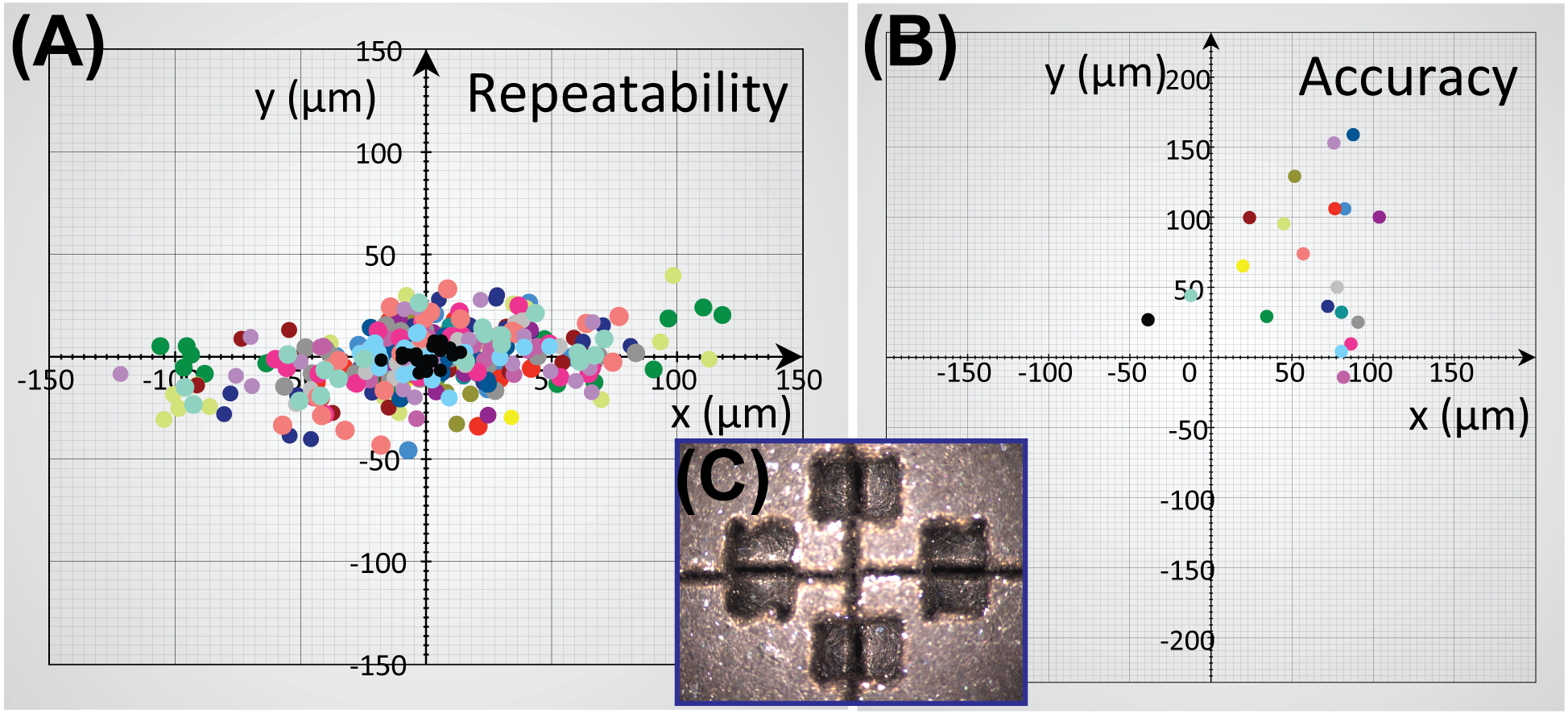

Supplement: Figure S2 — Characterization of Self-Alignment by Folding. Twenty independent Foldscopes were constructed out of 350 µm thick black cardstock and manually folded and unfolded twenty times each, with alignment measured after each assembly. The data was used to produce plots of (A) assembly repeatability (distribution of all 400 values, adjusted to give zero mean for each Foldscope) and (B) assembly accuracy (distribution of 20 mean values calculated per Foldscope) using (C) cross-hair alignment features on the optics and illumination stages. Note that the span of the data in both plots is less than the thickness of the paper used to construct the Foldscopes. Based on the data shown in the plots, assembly repeatability was assessed as the mean value of twice the standard deviation for each Foldscope (65 µm in X and 25 µm in Y), while assembly accuracy was assessed as the mean value of all trials (59 µm in X and 67 µm in Y). A higher skew in X-axis repeatability results from structurally distinct constraints implemented for the X- and Y-axes, while the assembly accuracy errors in both directions are consequences of the design which can be compensated by feature shifts in future designs. Note that the X and Y error bars for all measurements are 8.4 µm. (TIF) [file pone.0098781.s002.tif]

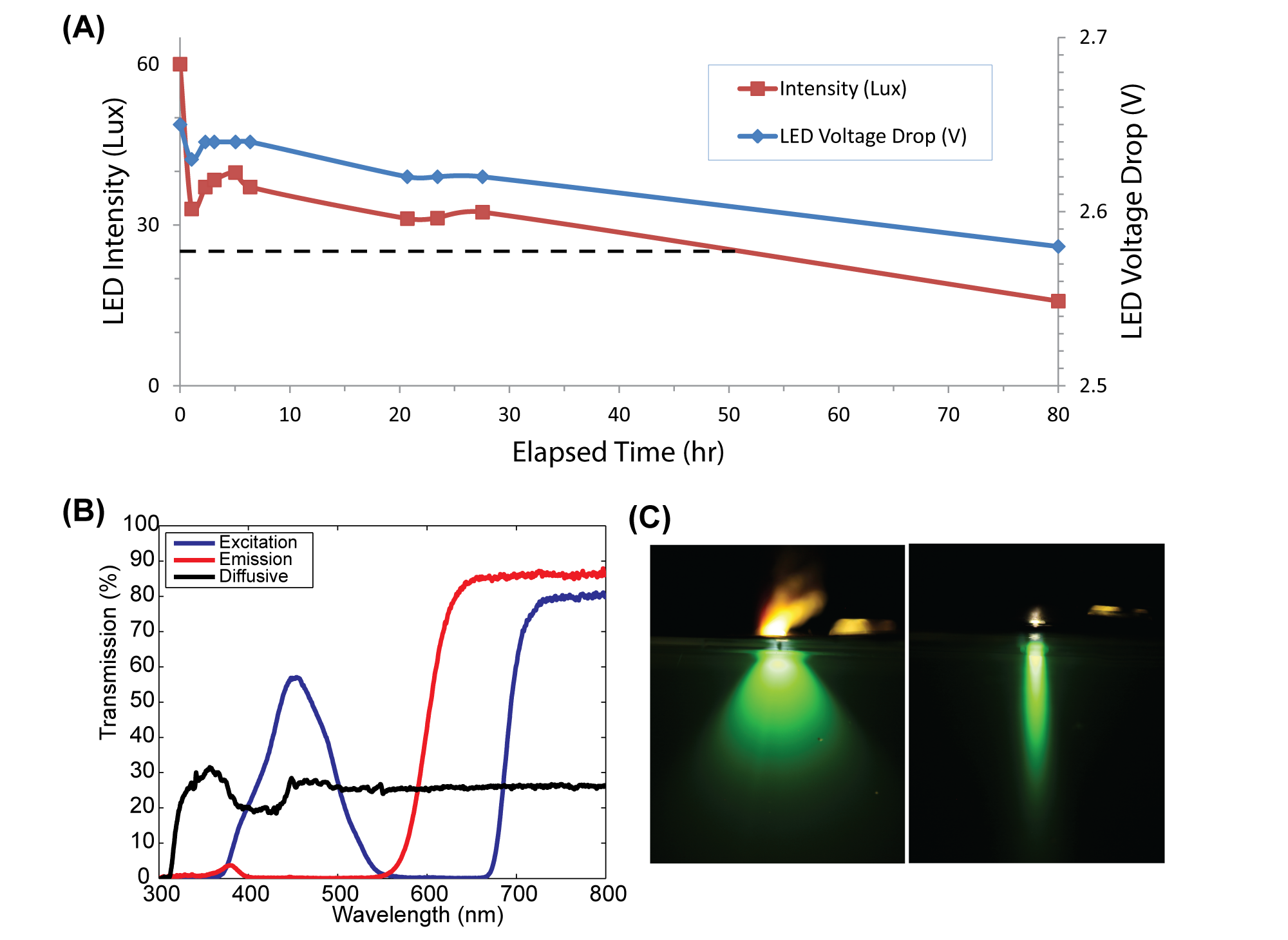

Supplement: Figure S3 — Component Characterization. (A) LED voltage and intensity versus time for a white LED (Avago HSMW-CL25) powered by a Duracell CR2032 battery with no resistor. (B) Filter transmission spectra of three Roscolux filters ― Tough Rolex diffuser (#111), Fire Red (#19), and Primary Blue (#80) ― measured with Ocean Optics Photo spectrometer USB4000. (C) Intensity profile of a white LED (Avago HSMW-CL25) as visualized in water with dissolved fluorescein. The left image is taken with the bare LED while the right image is taken with a condenser lens (2.4 mm borosilicate ball lens) placed adjacent to the LED in the optical path, demonstrating that a ball lens can be used to effectively collimate the light emitted by this LED. (TIF) [file pone.0098781.s003.tif]

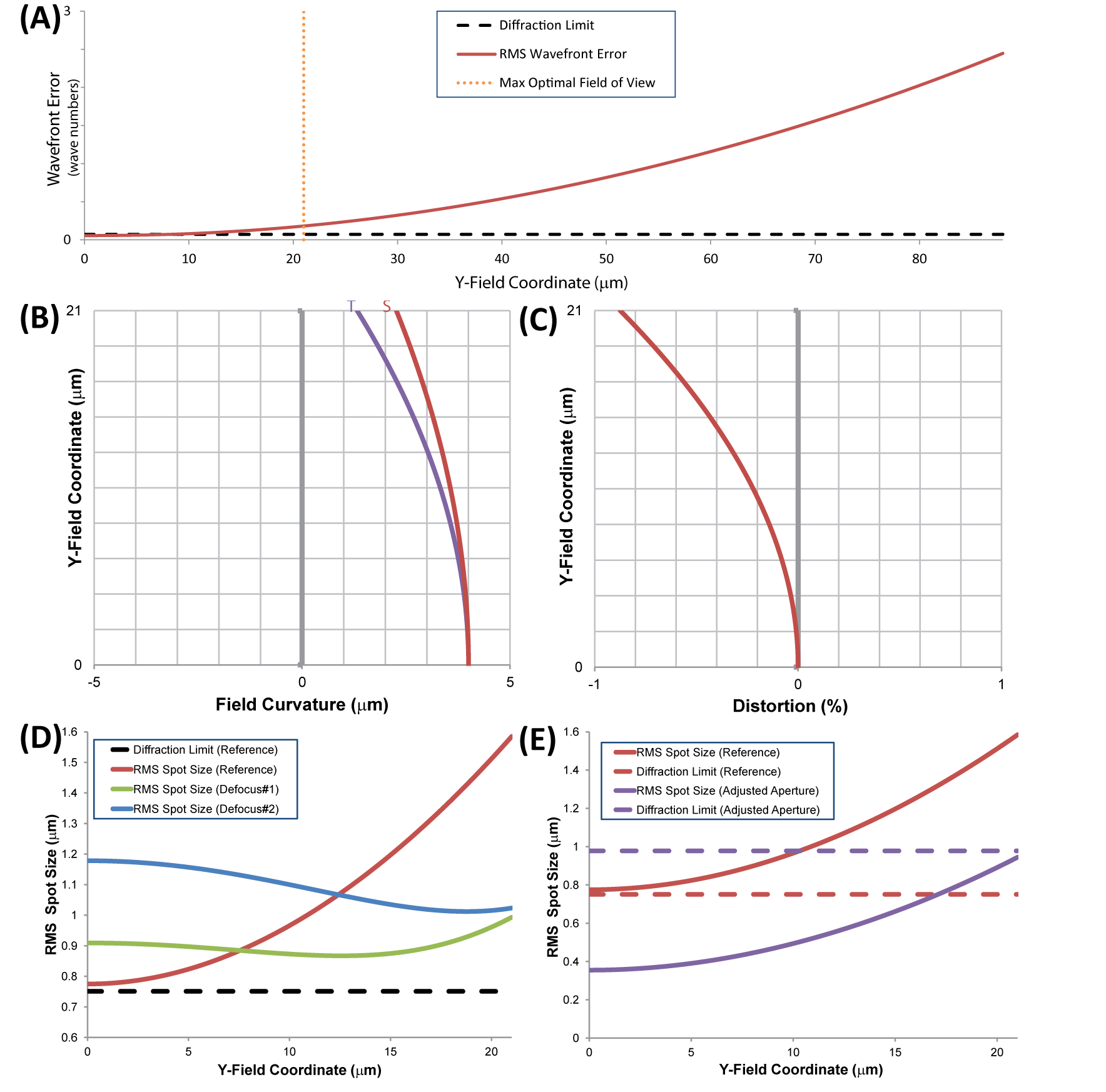

Supplement: Figure S4 — Numerical Modeling Characterization of Optimal Field of View. (A) Plot of Wavefront Error over the full field of view defined by the aperture for a 300 µm Sapphire ball lens with a 147 µm aperture. With increasing field coordinate, the Wavefront Error becomes very large and the image will be out of focus. An “optimal field of view” is defined at a field coordinate of 21 µm, where the Wavefront Error is approximately 1/5 wave number. (B,C) Plots of Field Curvature and Distortion over the optimal field of view. (D) Plot of RMS spot size over the optimal field of view depicting four cases: optimized solution treated as reference with zero defocus (red line), defocus of 3 µm (green line), defocus of 3 µm (green line), diffraction limit (dashed black line). The reference solution provides the best achievable resolution at the center of the field of view (approximately equal to the diffraction limit for this choice of aperture), while other plots show that increasing defocus moves the region of best resolution radially out from the center in an annular ring. (E) Plot of RMS spot size over the optimal field of view depicting optimal aperture predicted by analytical model (red lines) and adjusted aperture giving uniform RMS spot size over the field of view (purple lines). (TIF) [file pone.0098781.s004.tif]

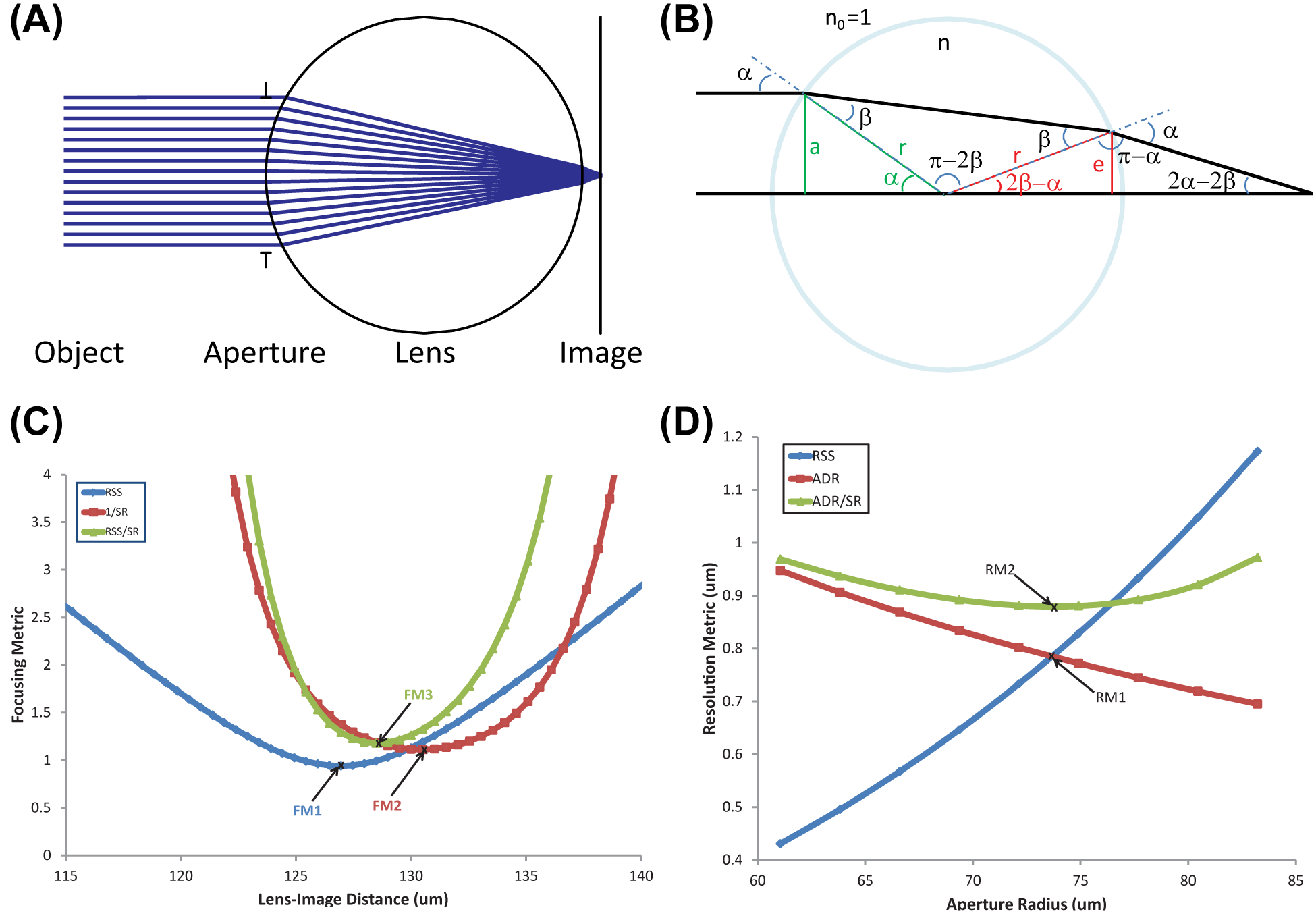

Supplement: Figure S5 — Diagrams and Plots for Numerical and Analytical Models. (A) Schematic of time-reversed Zemax model showing collimated light coming from an object at infinity, passing through aperture, and focused by the ball lens onto a focal point in the image plane. (B) Schematic of time-reversed model showing key parameters used in some derivations for the analytical model. (C) Plot of Focusing Metric versus Lens-Image Distance for λ = 0.55 µm, r = 150 µm, n = 1.517. This illustrates how focusing metrics FM1, FM2, and FM3 select different values for the optimal lens-image distance. (D) Plot of Resolution Metric versus Aperture Radius for λ = 0.55 µm, r = 150 µm, n = 1.517. This illustrates how resolution metrics RM1 and RM2 select nearly the same aperture radius but yield different values for resolution. (TIF) [file pone.0098781.s005.tif]

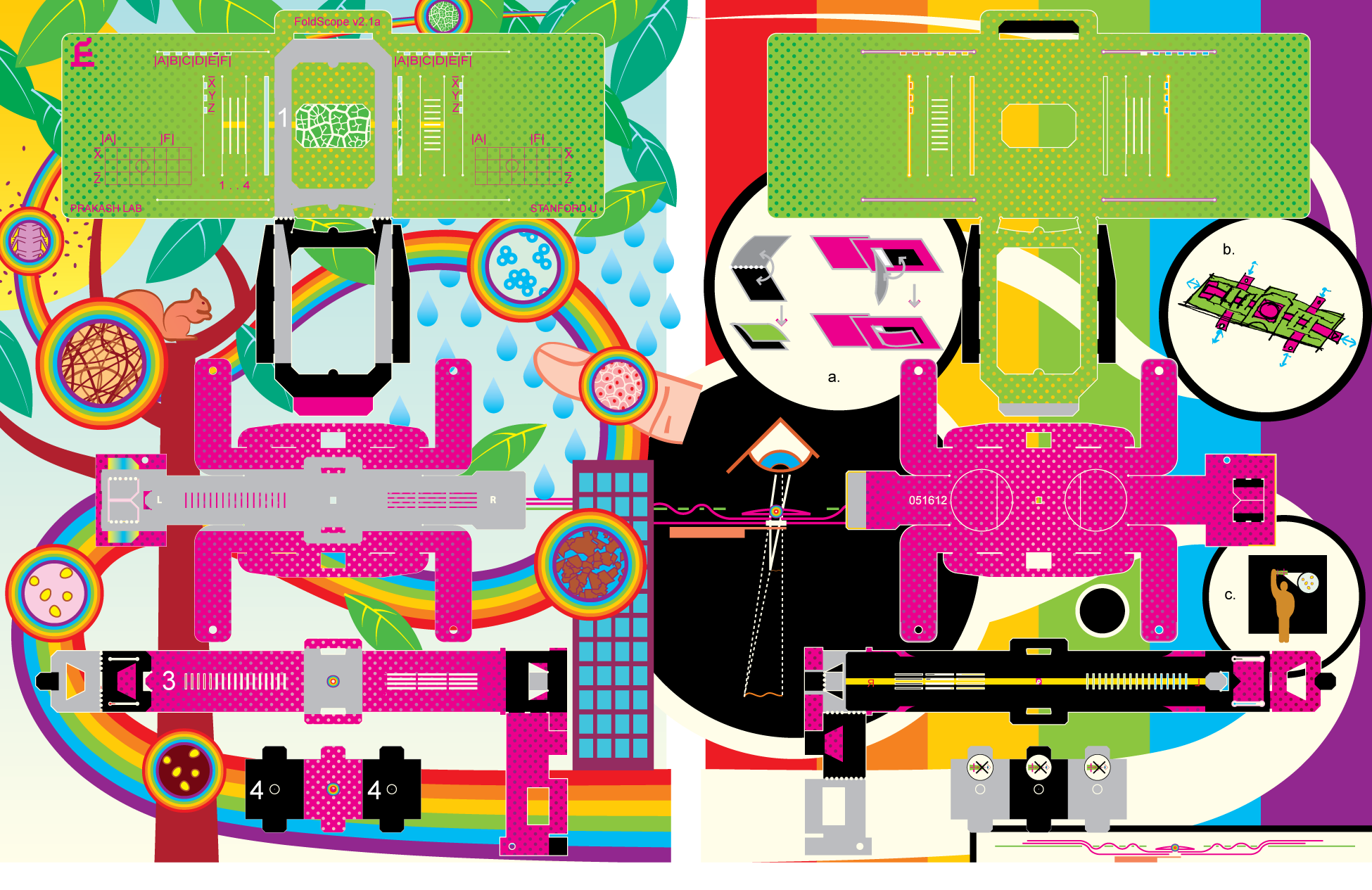

Supplement: Figure S6 — Artistic Layout of Foldscope Paper Components. Artistic version of Foldscope layout with integrated universal folding instructions based on color coding, where like colors are matched during the folding process to leave a single solid color in the final folded configuration. (TIF) [file pone.0098781.s006.tif]

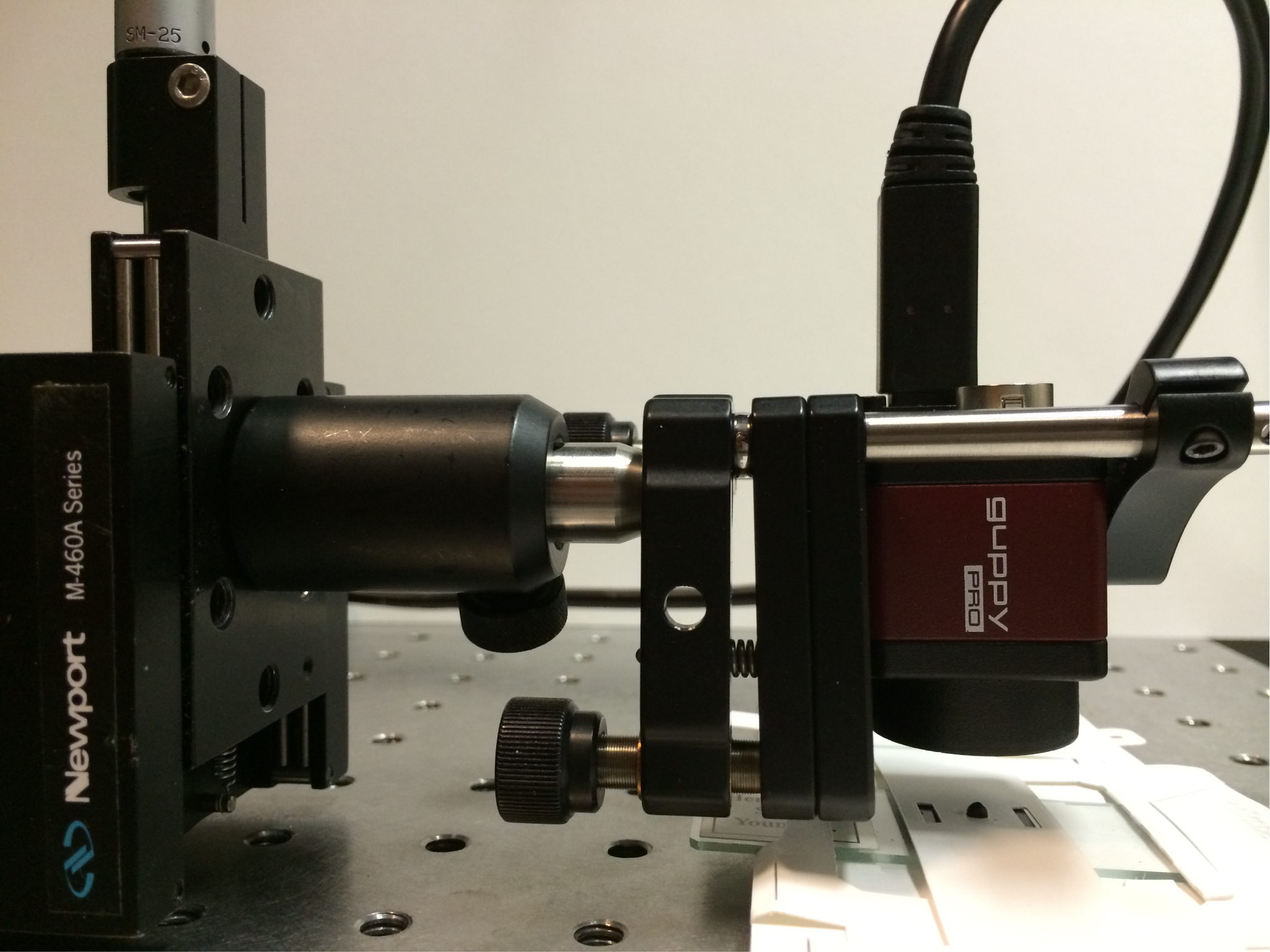

Supplement: Figure S7 — Foldscope Image Capture Setup for Resolution Metric. Picture of the experimental setup used to caputure images of the USAF 1951 resolution target viewed with the Foldscope. The data was taken using GUPPY Pro 503C scientific camera, with 2592×1944 pixels and pixel size 2.2×2.2 µm2. (TIF) [file pone.0098781.s007.tif]
