# Supplementary material for: Foldscope: Origami-Based Paper Microscope
Source: PLoS One. 2014 Jun 18;9(6):e98781. doi: 10.1371/journal.pone.0098781 (PMC4062392; doi:10.1371/journal.pone.0098781)
Supplement: Figure S8 — Quick Reference Guide for Foldscope Assembly. One-page handout to facilitate users in guided assembly of a Foldscope. (PDF) [file pone.0098781.s008.pdf]

FOLDSCOPE ASSEMBLY — QUICK REFERENCE GUIDE

STEP	INSTRUCTION	RESULT
1-2	REMOVE PARTS<br><br>FOLD "T" PART [1]	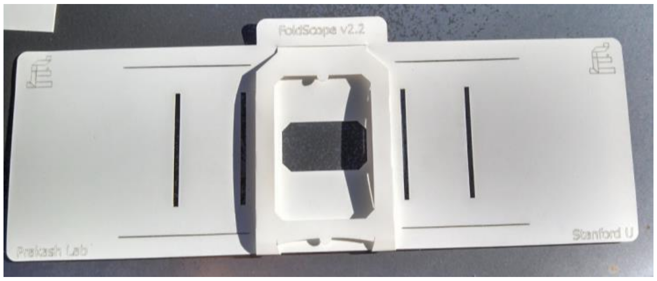
3-6	FOLD "L" PART [2]<br><br>MOUNT LENS	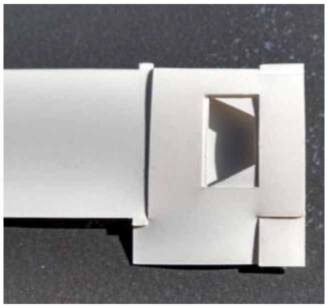 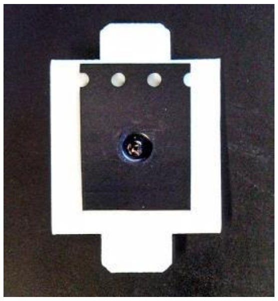
7-9	ASSEMBLE [1] + [2]<br><br>INSERT LENS	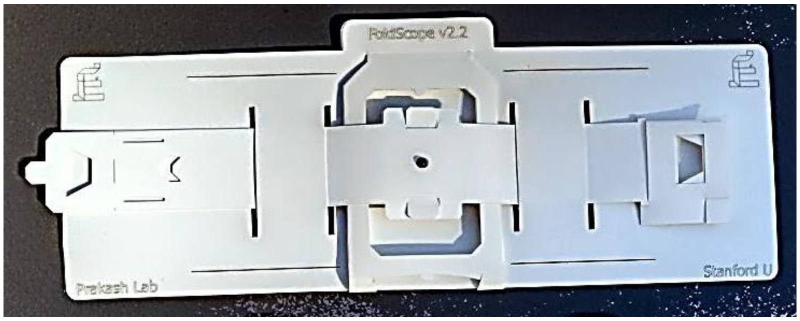
10-11	INSERT "TURTLE" PART [3]<br><br>ALIGN [2] + [3]	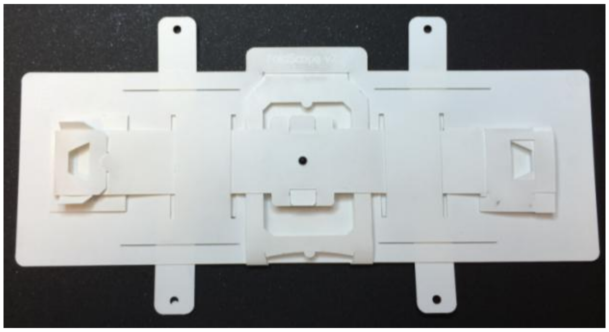
12-13	INSERT "TURTLE" LEGS<br><br>INSERT SLIDE AND VIEW!	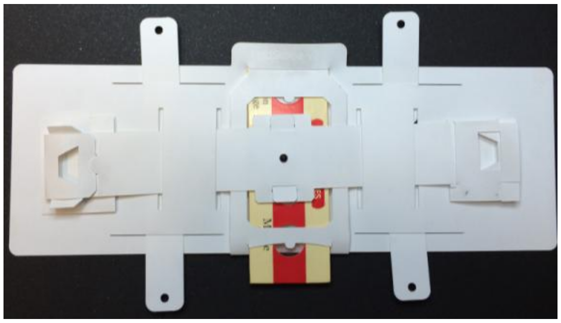
