# Supplementary material for: Foldscope: Origami-Based Paper Microscope
Source: PLoS One. 2014 Jun 18;9(6):e98781. doi: 10.1371/journal.pone.0098781 (PMC4062392; doi:10.1371/journal.pone.0098781)
Supplement: Text S1 — Supplementary information. Analytical expressions and derivations related to optical performance metrics. (DOCX) [file pone.0098781.s011.docx]

Supporting Information

Supplementary Materials included:

- Basic Expressions for Ball Lenses
- Analytical Model for a Foldscope in Brightfield
- Figures S1-S8
- Foldscope Assembly Video S1 (available online)
- Foldscope Drop Test Video S2 (available online)

Supplementary Materials:

**Basic Expressions for Ball Lenses.**

Using geometrical ray-tracing methods (see Figure S5B), the following expressions for optical properties of a ball lens can be derived under the paraxial approximation (sinθ=θ):

$EFL=nr/2(n-1)$ (Effective focal length)

$BFL=(2-n)r/2(n-1)$ (Back focal length)

$MAG=250mm/EFL$ (Magnification)

$FOV=na/2(n-1)$ (Field of View Radius)

$NA=2a(n-1)/nr$ (Numerical Aperture)

$DOF=\lambda/NA^{2}$ (Depth of Field)

These expressions are written in terms of the following parameters: ball radius (r), index of refraction (n), aperture radius (a), and incident wavelength (λ). See Table 2 for the functional forms of these expressions for an optimized aperture and for evaluation of the expressions at select parameter values.

**Analytical Model for an Foldscope in Brightfield.**

*Introduction.* The primary parameters for the Foldscope optical system are object-lens distance, lens-image distance, ball lens radius, aperture radius, ball lens index of refraction, and incident illumination wavelength. The goal of this optimization is to determine the aperture radius and object-lens distance that will provide the smallest resolvable feature size, or in other words, that will minimize resolution. The "object plane" and "image plane" are interchanged relative to the physical system so that the size of the "image" in the model corresponds to the size of the object in the physical system. This technique is physically valid since time reversal symmetry applies to optical systems. It is useful since the time-reversed system naturally lends itself to computation of the smallest achievable spot size at the focal point in the "image plane", which corresponds to the smallest resolvable object (i.e., the resolution) in the physical system.

In the description that follows, note that the terms "image" and "object" refer to their respective entities in the time-reversed system. Also note that the sign convention for the numerical and analytical models is that the object-lens distance is negative for real objects, and the lens-image distance is positive for real images. In both cases, the sign is reversed if the object or image is virtual.

In this analysis, the system is modeled for the special case of an object at infinity, which physically corresponds to collimated light emerging from the Foldscope. The models are used to obtain functional relationships for the aperture radius and resolution (as well as other calculated quantities) in terms the physical parameters of the ball lens (radius and index of refraction) and of the incident illumination (wavelength).

This model effectively optimizes image resolution based on one field point at the center of the field of view. The advantage of this approach is that the resolution in this region will be the best achievable resolution for a simple ball lens, providing a critical capability for some applications. The disadvantage is that the edges of the field of view will have significant defocus. A numerical model was used to evaluate the image quality over the whole field, to define an "Optimal field of view" with good image quality, and to suggest strategies for extending the region of optimal resolution (see figure S4 and discussion in Design Innovations section of the main text).

Two analytical models were developed ― one for each of the two resolution metrics, RM1 and RM2. These models predict the same exact functional forms for OAR and RES to a multiplicative constant, and these forms also show excellent agreement with the data from numerical modeling. The solution for these models is obtained in three steps. First, analytical expressions for OAR and RES are obtained in terms of the aberration coefficient of primary spherical aberration (s) for both resolution metrics. Second, an analytical expression is derived for the aberration coefficient, $s=s\left(n,r\right)$. Finally, these results are combined with an approximate expression for focal length $f=f\left(n,r\right)$ to yield the desired expressions, $OAR=OAR\left(\lambda,n,r\right)$ and $RES=RES\left(\lambda,n,r\right)$.

*Expressions from First Resolution Metric (RM1).* The first resolution metric is the absolute difference between the Airy Disk Radius (ADR) and RMS Spot Size (RSS),

$RM1\equiv\left|ADR-RSS\right|$ (Eq. 1)

This expression is minimized when,

$ADR=RSS$ (Eq. 2)

The Airy Disk Radius is defined as [1],

$ADR=1.22\cdot\lambda\cdot F$ (Eq. 3)

where F = the F/# or focal ratio, which is defined in terms of the focal length (f) and the aperture radius (a) as,

$F=\frac{f}{2\cdot a}$ (Eq. 4)

RMS Spot Size can be approximated by RMS blur radius (r_RMS_), which is given by [2-3],

$RSS\cong r_{RMS}=8F\cdot\left|S\right|\sqrt{\frac{1}{4}-\frac{\Lambda}{3}+\frac{\Lambda^{2}}{8}}$ (Eq. 5)

where F = focal ratio, S = peak aberration coefficient, and Λ = normalized longitudinal aberration. At best focus, Λ=1 and the approximate expression for RMS Spot Size simplifies to,

$RSS\cong\frac{4}{\sqrt{6}}F\cdot\left|S\right|$ (Eq. 6)

The peak aberration coefficient is given by,

$S=s\cdot a^{4}$ (Eq. 7)

where s = the aberration coefficient of primary spherical aberration. The normalized optimal aperture radius (nOAR=OAR/r) is found by substituting into (Eq. 2) from (Eq. 3), (Eq. 6), and (Eq. 7) and solving for a/r. This yields,

$nOAR_{RM1}=\frac{k_{1,RM1}}{r}\cdot\left(\frac{\lambda}{\left|s\right|}\right)^{1/4}$, $k_{1,RM1}=\left(\frac{1.22\cdot\sqrt{6}}{4}\right)^{1/4}\cong0.9297$ (Eq. 8)

The corresponding resolution is found by substituting a=OAR into the expression for Airy Disk Radius (or into the expression for RMS Spot Size). Substituting from (Eq. 4) and (Eq. 8) into (Eq. 3), the resolution for the first resolution metric is,

$RES_{RM1}=k_{2,RM1}\cdot f\cdot\left(\lambda^{3}\cdot\left|s\right|\right)^{1/4}$, $k_{2,RM1}=\left(\frac{1.22^{3}}{4\sqrt{6}}\right)^{1/4}\cong0.6561$ (Eq. 9)

*Expressions from Second Resolution Metric (RM2).* As stated previously, the second resolution metric is the Airy Disk Radius (ADR) divided by the Strehl Ratio (SR). This is minimized when its derivative with respect to the aperture radius equals zero,

$\frac{\partial}{\partial a}\left(\frac{ADR}{SR}\right)=0$ (Eq. 10)

Applying the quotient rule leads to the following equivalent condition:

$SR\cdot\frac{\partial ADR}{\partial a}=ADR\cdot\frac{\partial SR}{\partial a}$ (Eq. 11)

The Strehl Ratio is approximated by a simple empirical expression given by Mahajan [4],

$SR=e^{-\left(2\pi\omega_{s}/\lambda\right)^{2}}$ (Eq. 12)

where ω_s_ = RMS wavefront error at the best focus. The RMS wavefront error due to spherical aberration at best focus is given by [2-3],

$\omega_{s}=\frac{S}{\sqrt{180}}$ (Eq. 13)

where S = peak aberration coefficient as given by (Eq. 7). Substituting (Eq. 7) and (Eq. 13) into (Eq. 12), the Strehl ratio can be written as,

$SR=e^{-C_{1}\cdot a^{8}}$, $C_{1}=\left(\frac{\pi\cdot s}{3\sqrt{5}\cdot\lambda}\right)^{2}$ (Eq. 14)

Using (Eq. 3) and (Eq. 4), the Airy Disk Radius can be written as,

$ADR=\frac{C_{2}}{a}$, $C_{2}=\frac{1.22\cdot\lambda\cdot f}{2}$ (Eq. 15)

The expression for the second resolution metric is therefore,

$\frac{ADR}{SR}=\frac{C_{2}}{a}\cdot e^{C_{1}\cdot a^{8}}$ (Eq. 16)

Taking the derivatives of (Eq. 14) and (Eq. 15) with respect to the aperture radius yields,

$\frac{\partial SR}{\partial a}=-8C_{1}\cdot a^{7}\cdot e^{-C_{1}\cdot a^{8}}$ (Eq. 17)

$\frac{\partial ADR}{\partial a}=\frac{-C_{2}}{a^{2}}$ (Eq. 18)

Substituting (Eq. 14), (Eq. 15), (Eq. 17), and (Eq. 18) into (Eq. 11) and solving for a/r gives the following expression for the normalized optimal aperture radius (nOAR=OAR/r) corresponding to the second resolution metric,

$nOAR_{RM2}=\frac{k_{1,RM2}}{r}\cdot\left(\frac{\lambda}{\left|s\right|}\right)^{1/4}$, $k_{1,RM2}=\left(\frac{3\sqrt{10}}{4\pi}\right)^{1/4}\cong0.9321$ (Eq. 19)

The corresponding resolution is found by substituting a=OAR into the expression for ADR/SR. Substituting (Eq. 19) into (Eq. 16), and eliminating C_1_ and C_2_ using (Eq. 14) and (Eq. 15), the resolution for the second resolution metric is,

$RES_{RM2}=k_{2,RM2}\cdot f\cdot\left(\lambda^{3}\cdot\left|s\right|\right)^{1/4}$, $k_{2,RM2}=1.22\cdot\left(\frac{\pi}{12}\right)^{1/4}\cdot\left(\frac{e}{10}\right)^{1/8}\cong0.7415$ (Eq. 20)

*Comparison of RM1 Model to RM2 Model.* Note that the two resolution metrics RM1 and RM2 yield identical functional forms for nOAR and RES. Compare (Eq. 8) to (Eq. 19) and (Eq. 9) to (Eq. 20). Their predicted values for k_1_ differ only by 0.26%, indicating excellent agreement for the size of the optimal aperture. Since RM2 is a more conservative metric that includes the Strehl Ratio, it is not surprising that it predicts coarser resolution, with k_2_ 11.5% larger than the RM1 model.

Also note that the analyses of the two models are perfectly general up to here, within the limits of the approximations in (Eq. 6) for RMS Spot Size and (Eq. 12) for Strehl Ratio. Therefore, the expressions for nOAR and RES may be applied to other, more complex systems (not just ball lenses) to determine values for optimal aperture radius and resolution, given expressions for the aberration coefficient (s) and the focal length (f) for such a system.

*Expression for aberration coefficient for primary spherical aberration.* This system consists of two optical surfaces and has a circular aperture concentrically located on the first surface. To find an expression for the aberration coefficient (s_i_) for the i^th^ surface of a system, the wave aberration (W) is first calculated for that surface as the optical path difference between the chief ray and a marginal ray. The aberration coefficient (s) is then obtained as the coefficient of the term with aperture radius to the 4^th^ power. From the derivation by Mahajan [4], the resulting expression for the aberration coefficient of the i^th^ surface is,

(Eq. 21a)

where n_i_, n_i_' are the refractive indices of the media before and after the surface; L_i_, L_i_' are the object-lens distance and the lens-image distance for the i^th^ surface; and R_i_ is the ball lens radius (positive if the arc is centered to the right of the surface, and negative if the arc is centered to the left of the surface). The following alternate forms,

(Eq. 21b)

(Eq. 21c)

can be obtained by eliminating L_i_ and L_i_', respectively, from (Eq. 21a) using the Gaussian imaging equation [1],

(Eq. 22)

Using subscripts to denote the surface, the indices of refraction of this system are n_1_=n_2_'=1 and n_1_'=n_2_=n, where n = index of refraction of the ball lens. The radii of the respective surfaces are R_1_=-R_2_=r, where r = the radius of curvature of the ball lens. In this model, the object is assumed to be at infinity (L_1_= infinity), so L_1_' equals the focal length (f_1_). Substituting the values for the first surface in (Eq. 22) and solving for the lens-image distance (L_1_') gives,

$L'_{1}=f_{1}=\frac{n\cdot r}{n-1}$ (Eq. 23)

Since the image plane of the first surface serves as the object plane of the second surface, and the two surfaces are separated by twice the ball lens radius, the object-lens distance for the second surface can be written as,

$L_{2}=L'_{1}-2r$ (Eq. 24)

Substituting (Eq. 23) into (Eq. 24) gives,0

$L_{2}=\frac{r\cdot\left(2-n\right)}{n-1}$ (Eq. 25)

Writing out (Eq. 21c) for i=1 and i=2, and substituting in the corresponding values for these surfaces gives,

$s_{1}=-\frac{n-1}{8n^{2}r^{3}}$ (Eq. 26)

$s_{2}=-\frac{n\cdot\left(n-1\right)\cdot\left(2n-1\right)}{8r^{3}\cdot\left(2-n\right)^{3}}$ (Eq. 27)

The total aberration coefficient is the sum of the aberration coefficients from the two surfaces, with the coefficient for the second surface weighted by the fourth power of the ratio of the effective aperture radius on the second surface (e) to the aperture radius on the first surface (a),

$s=s_{1}+\left(\frac{e}{a}\right)^{4}\cdot s_{2}$ (Eq. 28)

From the ray tracing diagram in figure S5B, it is evident that the ratio e/a can be written as,

$\frac{e}{a}=\frac{r\cdot\sin\left(2\beta-\alpha\right)}{r\cdot\sin\left(\alpha\right)}$ (Eq. 29)

where α = angle of incidence of incoming collimated light, β = angle of incidence of light inside the glass, and r = radius of the ball lens. A relation between the angles α and β is provided by Snell's Law,

$\sin\left(\alpha\right)=n\cdot\sin\left(\beta\right)$ (Eq. 30)

Applying the paraxial approximation sinθ=θ to (Eq. 29) and (Eq. 30) and combining the results gives the following approximate expression for e/a,

$\frac{e}{a}\cong\frac{2\beta}{\alpha}-1\cong\frac{2-n}{n}$ (Eq. 31)

Substituting (Eq. 26), (Eq. 27), and (Eq. 31) in (Eq. 28) gives the following expression for the aberration coefficient,

$s=-\frac{\left(n-1\right)\cdot\left[n+\left(2-n\right)\cdot\left(2n-1\right)\right]}{\left(2r\cdot n\right)^{3}}$ (Eq. 32)

While this expression was derived independently as described above, it is equivalent to an expression for the Sidel coefficient S_I_ for a thick lens recently published in Applied Optics by Miks and Novak [5].

*Expressions for OAR=OAR(λ,n,r) and RES=RES(λ,n,r).* Now that an expression has been obtained for the aberration coefficient s(n,r), all that is needed to find OAR(λ,n,r) and RES(λ,n,r) is an expression for the focal length f(n,r). This is given by the Lensmaker's equation [1],

$\frac{1}{f}=\left(n-1\right)\cdot\left[\frac{1}{R_{1}}-\frac{1}{R_{2}}+\frac{\left(n-1\right)\cdot d}{n\cdot R_{1}\cdot R_{2}}\right]$ (Eq. 33)

where n = index of refraction of the ball lens; R_1_,R_2_ = radii of curvature of the respective surfaces; and d = spacing between the surfaces. Substituting R_1_=-R_2_=r and d=2r, the effective focal length (EFL) for a ball lens is,

$f=EFL=\frac{r\cdot n}{2\left(n-1\right)}$ (Eq. 34)

Since the resolution metrics RM1 and RM2 both yielded the same functional form for nOAR and RES in terms of s(n,r) and f(n,r), their final results will also be the same within a multiplicative constant. Substituting (Eq. 34) and (Eq. 32) into (Eq. 8) and (Eq. 9) for RM1 and into (Eq. 19) and (Eq. 20) for RM2, the final expressions for normalized optimal aperture radius (nOAR) and resolution (RES) are,

$nOAR=k_{1}\cdot\left(\frac{8\lambda\cdot n^{3}}{r\cdot\left(n-1\right)\cdot\left[n+\left(2-n\right)\cdot\left(2n-1\right)\right]}\right)^{1/4}$ (Eq. 35)

$RES=k_{2}\cdot\left(\frac{\lambda^{3}\cdot r\cdot n\cdot\left[n+\left(2-n\right)\cdot\left(2n-1\right)\right]}{128\left(n-1\right)^{3}}\right)^{1/4}$ (Eq. 36)

with the following values for the multiplicative constants k_1_ and k_2_ as given by (Eq.8), (Eq.9), (Eq.19), and (Eq.20),

$k_{1,RM1}=\left(\frac{1.22\cdot\sqrt{6}}{4}\right)^{1/4}\cong0.9297$ (Eq. 37)

$k_{2,RM1}=\left(\frac{1.22^{3}}{4\sqrt{6}}\right)^{1/4}\cong0.6561$ (Eq. 38)

$k_{1,RM2}=\left(\frac{3\sqrt{10}}{4\pi}\right)^{1/4}\cong0.9321$ (Eq. 39)

$k_{2,RM2}=1.22\cdot\left(\frac{\pi}{12}\right)^{1/4}\cdot\left(\frac{e}{10}\right)^{1/8}\cong0.7415$ (Eq. 40)

**Supplementary References:**

1. Hecht E (1987) Optics, 2nd ed. Addison Wesley.
2. Sacek V (2006) Amateur Telescope Optics. http://www.telescope-optics.net. Accessed 1 May 2014.
3. Mahajan VN (2011) Aberration Theory Made Simple, 2nd ed. Bellingham, Washington: SPIE Press.
4. Mahajan VN (1998) Optical Imaging and Aberrations: Wave Diffraction Optics. SPIE-International Society for Optical Engineering Press. 450 p.
5. Miks A, Novak J (2012) Third-order aberration coefficients of a thick lens. Applied Optics, 51(33): 7883-7886. doi: http://dx.doi.org/10.1364/AO.51.007883.
